# The antifungal potential of (*Z*)-ligustilide and the protective effect of eugenol demonstrated by a chemometric approach

**DOI:** 10.1038/s41598-019-45222-y

**Published:** 2019-06-19

**Authors:** Alice M. S. Rodrigues, Véronique Eparvier, Guillaume Odonne, Nadine Amusant, Didier Stien, Emeline Houël

**Affiliations:** 1 Sorbonne Université, CNRS, Laboratoire de Biodiversité et Biotechnologie Microbienne, LBBM, Observatoire Océanologique, 66650 Banyuls-sur-mer, France; 2 CNRS, Institut de Chimie des Substances Naturelles, UPR2301, Université Paris-Saclay, 91198 Gif-sur-Yvette, France; 3 Laboratoire Ecologie, Evolution, Interactions des Systèmes Amazoniens (LEEISA), CNRS, Université de Guyane, IFREMER, 97300 Cayenne, France; 4 CIRAD, UMR EcoFoG, AgroParisTech, CNRS, INRA, Université des Antilles, Université de Guyane, 97300 Cayenne, France; 5 CNRS, UMR EcoFoG, AgroParisTech, Cirad, INRA, Université des Antilles, Université de Guyane, 97300 Cayenne, France

**Keywords:** Metabolomics, Antifungal agents

## Abstract

Mankind is on the verge of a postantibiotic era. New concepts are needed in our battle to attenuate infectious diseases around the world and broad spectrum plant-inspired synergistic pharmaceutical preparations should find their place in the global fight against pathogenic microorganisms. To progress towards the discovery of potent antifungal agents against human pathologies, we embarked upon developing chemometric approach coupled with statistical design to unravel the origin of the anticandidal potential of a set of 66 essential oils (EOs). EOs were analyzed by GC-MS and tested against *Candida albicans* and *C*. *parapsilosis* (Minimal Inhibitory Concentration, MIC). An Orthogonal Partial Least Square (OPLS) analysis allowed us to identify six molecules presumably responsible for the anticandidal activity of the oils: (*Z*)-ligustilide, eugenol, eugenyl acetate, citral, thymol, and β-citronellol. These compounds were combined following a full factorial experimental design approach in order to optimize the anticandidal activity and selectivity index (SI = IC_50_(MRC_5_ cells)/MIC) through reconstituted mixtures. (*Z*)-Ligustilide and citral were the most active compounds, while (*Z*)-ligustilide and eugenol were the two main factors that most contributed to the increase of the SI. These two terpenes can, therefore, be used to construct bioinspired synergistic anticandidal mixtures.

## Introduction

Microbial pathogen chemoresistance has become a worrying worldwide phenomenon^1,2^ and the global mortality of patients suffering from microbial infections is increasing regularly. Resistance to azoles and cross-resistances to azoles and echinocandins are now emerging in medically relevant fungi^3,4^. In fact, it seems that pathogens may be more indomitable than imagined, and even the WHO, alongside several authors, is indicating that humanity is on the verge of a postantibiotic era^5,6^. Moreover, it is now demonstrated that antibiotic resistance is a natural and ancient phenomenon, even described in pristine environments without exposure to human antimicrobial agents. Inadequate use of antimicrobials may also promote the selection of pre-existing resistance elements^7,8^. Therefore, even the well-reasoned use of antibacterial and antifungal compounds may be insufficient to prevent the acquisition of resistance, and new concepts and techniques are necessary to manage microbial pathologies.

Meanwhile, it is common knowledge that synergism, or at least combination therapies, is indispensable in limiting the risk of development of chemoresistant pathogens. This fact as well as the underlying mechanisms is thoroughly explored to discover new treatment strategies^9,10^. In this perspective, we may have much to learn from studying the natural world, in particular synergies in plant defensive traits. Indeed, some examples highlight that chemical defense against biotic and abiotic stress may rely on synergy^11–13^, even if these phenomena do not lead to a general rule^14^. Interestingly, this concept may also be extended to the combination of exogenous chemicals from various plant sources used by animal species to defend themselves, a phenomenon defined as “acquired combinatorial chemical defense”^15^. Eventually, biomimetic synergistic systems may provide new solutions to combat human pathogens.

Plant volatile organic compounds serve as chemical answers to the pressure from predators and pathogens and in conspecific and mutualistic interactions. They act through various modes of action linked to the structural and functional diversity of their components^12,16^. Due to these characteristics, multifaceted and versatile mixtures can find numerous applications. Alone or in combination with clinical drugs the mixtures can exhibit remarkable activities against various pathogenic microorganisms^17–19^ and synergistic effects between their constituents have also been demonstrated^20–22^. They could, therefore, represent one solution to fight microbial resistance. More particularly, synergy is a composite concept, simultaneously uniting interactions between inactive molecules to create active mixtures and isolated active compounds leading to even more active associations, the second point being the focus of our research.

The current leading method to evaluate complex mixtures is metabolomics^23^. This field consists of the global analysis of small molecules characterizing a biological system, while traditional bioguided fractionation does not consider synergistic effects^24^. When associated with chemometrics, metabolomics will guide the identification given activity markers^25,26^. Maree *et al*.^27^ recently explored the correlation of antimicrobial activity and chemical composition of various essential oils by GC-MS and statistics (Orthogonal Partial Least Square Discriminant Analysis, OPLS-DA). Their work nominated eugenol as the most potent antibacterial compound in the dataset.

The next stage would consist in evaluating interactions between selected combinations of compounds, notably through the use of statistical design, which consists of systematically investigating the effect of independent variables on a studied response. This technique was used to evaluate the influence of extraction solvent^28^ or mixture composition^29^ on the antimicrobial activity of plant extracts. Interactions between the four major constituents of *Thymus vulgaris* essential oil were also studied using statistical models to evaluate their insecticidal activity, highlighting synergy between thymol and *p*-cymene in a binary mixture^30^.

Because our group previously highlighted that several EOs antimicrobial potential originated from synergy^22,31^, we embarked upon characterizing 66 EOs in order to better understand their antifungal activity and eventually propose optimized antifungal mixtures using a full factorial experimental design.

## Results

### Essential oils anticandidal activity and composition

The antimicrobial activities of 66 essential oils off commercial or laboratory origin were evaluated against *C*. *albicans* and two strains of *C*. *parapsilosis*. The results are provided in the Supporting Information (Table S1). Antimicrobial activity was represented through a score ranking the global antifungal activity (Antifungal Activity Score, AAS). The scores ranged from 0 to 12, with 13 EOs having antifungal scores ≥ 5 (Table 1). GC/MS analyses performed on all EOs allowed for the detection of 343 different compounds, distinguished by a compound code according to their retention time and mass spectra. Major compounds identified among the most active EOs are also listed in Table 1.

**Table 1:** Antifungal activity score (AAS), botanical identification and main compounds identified for the most active essential oils.

Botanical identification*	Antifungal Activity Score (AAS)	Main compounds
*Backhousia citriodora* (c)	7	(*E*)-Citral (geranial, 53.1%); (*Z*)-Citral (neral, 43.3%)
*Cymbopogon citratus* (c)	10	(*E*)-Citral (geranial, 47.6%); (*Z*)-Citral (neral, 34.4%)
*Cymbopogon citratus* (l, sample 1)	9	(*E*)-Citral (geranial, 50.4%); (*Z*)-Citral (neral, 34.6%)
*Cymbopogon citratus* (l, sample 2)	8	(*E*)-Citral (geranial, 49.5%); (*Z*)-Citral (neral, 32.3%)
*Leptospermum petersonii* (c)	5	(*E*)-Citral (geranial, 32.6%); (*Z*)-Citral (neral, 29.8%)
*Levisticum officinale* (c)	11	(*Z*)-Ligustilide (79.8%)
*Melissa officinalis* (c)	5	(*E*)-Citral (geranial, 28.4%); (*Z*)-Citral (neral, 18.8%)
*Pelargonium graveolens* var2 (c)	5	(−)-Citronellol (22.8%)
*Pimenta racemosa* (l)	5	Eugenol (57.4%)
*Protium heptaphyllum* (l)	10	Limonene (90.0%)
*Sphagneticola trilobata* (l)	5	α-Pinene (77.1%)
*Syzygium aromaticum* (c)	8	Eugenol (85.7%); Eugenyl acetate (11.2%)
*Thymus vulgaris* var1 (c)	12	Thymol (56.8%)

The antifungal activity score is calculated based on the measured minimum inhibitory concentrations (MICs), according to the following scheme: >512 µg/mL = 0; 512 µg/mL = 1; 256 = 2; 128 = 3; 64 = 4. The final score is obtained by adding together the values obtained for each fungal strain.

*(c): commercial; (l): laboratory.

### Identification of putative antifungal activity markers by OPLS

**Figure 1:**
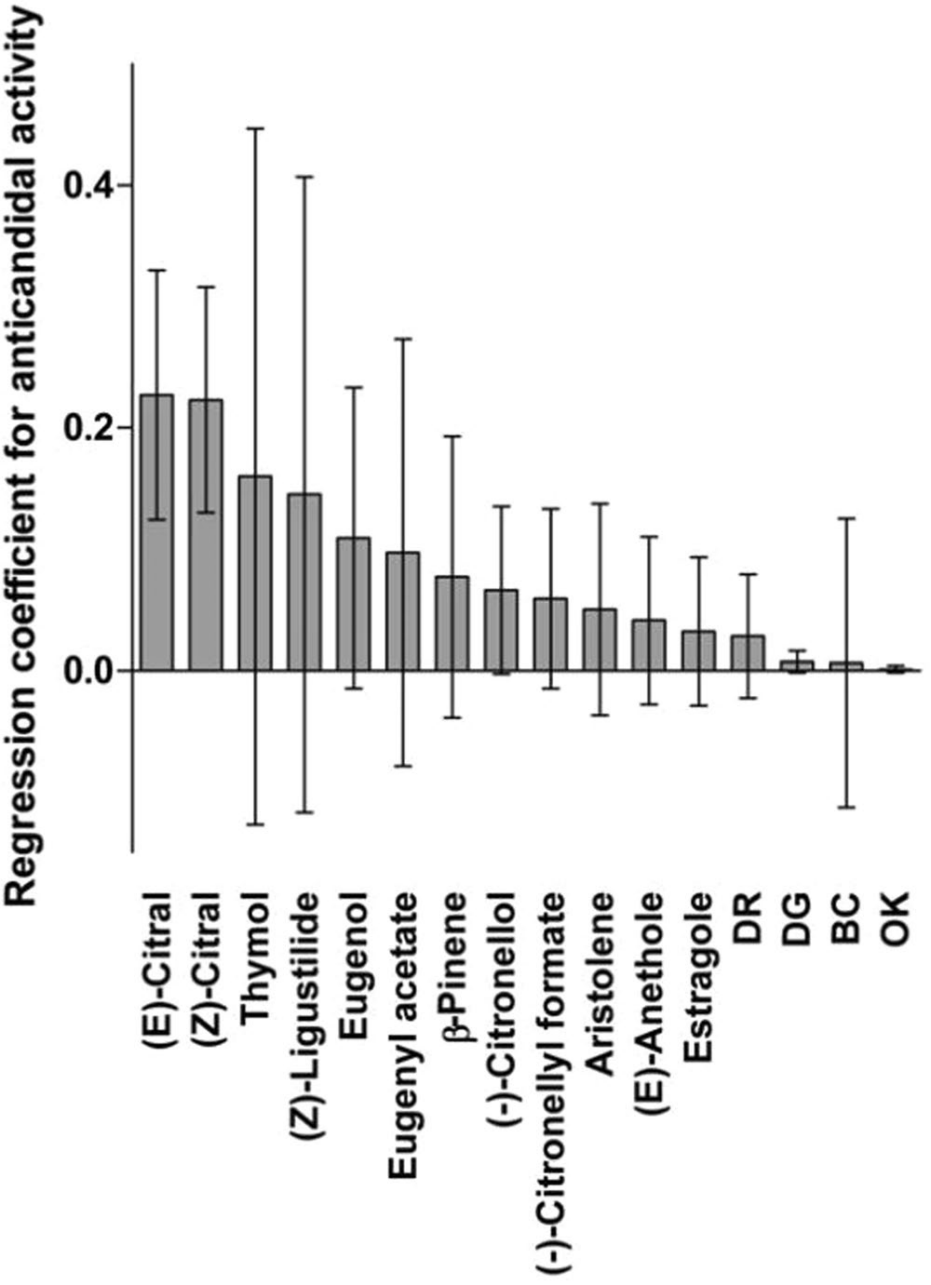
Coefficient-plot of the OPLS analysis limited to compounds accounting for more than 9% in at least one EO (60 metabolites). Only compounds with positive regression coefficient are reported.

In a second step, the regression coefficients were calculated by an OPLS model for all compounds accounting for at least 9% in one EO (60 compounds), in relation to EOs anticandidal activity. The predictive accuracy of the model was characterized by a R^2^ value of 0.67 and a Q^2^ value of 0.34. The compounds that most contributed to the antifungal activity are represented in Fig. 1.

All putative markers were tested individually. Whereas β-pinene had a positive regression coefficient, this compound was not considered further because it was found to be inactive according to endpoint criteria proposed by Cos *et al*. and Gertsch *et al*.^32,33^, exhibiting a MIC > 64 µg/mL when concurrently tested as a pure compound. The positive coefficient of β-pinene in the OPLS model coefficient-plot evidently originated from its presence in a large relative proportion (6.7 and 14.9%) in moderately active *Pimenta racemosa* EOs (antifungal scores of 4–5), along with approximately 60% of eugenol. β-Pinene was also present in many inactive essential oils, although always in a proportion below 6%. Overall, this explains why its regression coefficient was positive, and why β-pinene was excluded from the list of active metabolites.

Eventually, the seven compounds identified as the most influential for this model were (*E*)-citral (Variable Importance for the Projection 3.12), (*Z*)-citral (VIP 3.11), thymol (VIP 0.96), (*Z*)-ligustilide (VIP 0.89), eugenol (VIP 1.57), eugenyl acetate (VIP 1.07), and (−)-citronellol (VIP 1.42). OPLS analysis highlighted several of the major compounds in active EOs, but also relatively rare compounds (present in only one active EO) such as thymol or (*Z*)-ligustilide. In contrast, other compounds such as α-pinene or limonene did not correlate to the EOs antifungal activity because they were also present in some of the inactive EOs. Whereas thymol and ligustilide had VIP values slightly lower than 1, they were selected for further analysis^34,35^. Their role as putative marker was confirmed by their interesting antifungal potential when tested individually. All selected compounds had AAS ranging from 3 (eugenol) to 9 ((*Z*)-ligustilide).

### Combination effects of compound mixtures on anticandidal activity

Citral was commercially available as a mixture of (*Z*) and (*E*) isomers. Since the contribution of both isomers seemed identical, the mixture of (*Z*) and (*E*)-citral was used for the rest of the work under the name citral. The effect of the six putative antifungal markers citral (Ci), thymol (Th), (*Z*)-ligustilide (Li), eugenol (Eu), eugenyl acetate (EA), and (−)-citronellol (–C) alone or in combination with the others was measured using a full factorial design of experiment (DOE). A fractional DOE appeared inappropriate as it was difficult to omit a priori one interaction effect or the other while a full factorial designed required only 128 experiments (64 in duplicate).

**Figure 2:**
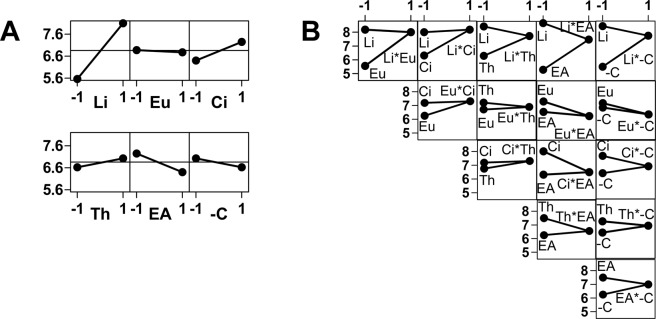
(**A**) Main effect plot for Antimicrobial Activity Score (AAS, ordinates). Li: (*Z*)-Ligustilide; Eu: Eugenol; Ci: Citral; Th: Thymol; EA: Eugenyl acetate; -C: (−)-Citronellol. Abscissa = –1: average AAS for mixtures that do not contain the compound; Abscissa = 1: average AAS for mixtures containing the compound. (**B**) Interaction plot for Antimicrobial Activity Score. Abscissa = –1: the two dots represent the average AAS for mixtures that contain the cited compound and do not contain the other; Abscissa = 1: average AAS for mixtures that contain both compounds. In this representation, two positive slopes indicate synergism, and two negative slopes denote antagonism. Otherwise, the effects are additive.

The effect of factors and interaction plots for the antifungal activity calculated from the DOE are presented in Fig. 2. (Z)-ligustilide is the strongest antimicrobial compound (*p* < 0.0001). Among the 64 combinations tested, for which the antifungal scores ranged from 0 (control) to 11 (Li:Eu 50:50), all the mixtures containing ligustilide exhibited antifungal scores ≥7, with an average antifungal score of 8.1, whereas the mixtures that did not contain (*Z*)-ligustilide exhibited an average antifungal score of 5.6. Citral also exhibited a significant antifungal effect (*p* 0.013). Moreover, the full interaction plot indicates that a slight positive interaction effect exists between citral and (*Z*)-ligustilide (average antifungal score of 8.2), thus demonstrating a synergistic effect of these two compounds.

### Combination effects of compounds mixtures on selectivity index

**Figure 3:**
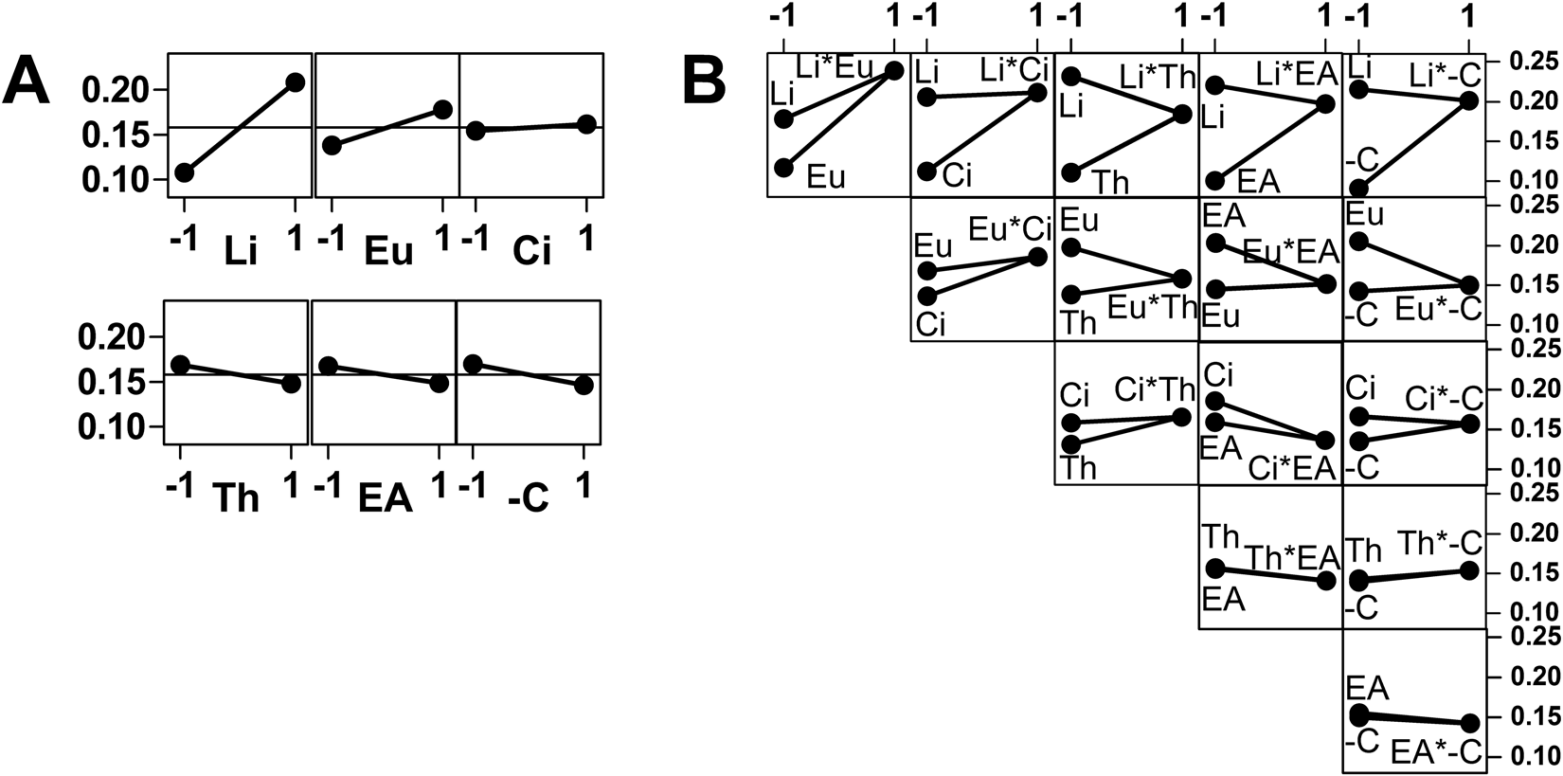
(**A**) Main effect plot for selectivity index (SI, ordinates). Li: *(Z*)-Ligustilide; Eu: Eugenol; Ci: Citral; Th: Thymol; EA: Eugenyl acetate; -C: (−)-Citronellol. Abscissa = −1: average SI for mixtures that do not contain the compound; Abscissa = 1: average SI for mixtures containing the compound. (**B**) Interaction plot for selectivity index. Abscissa = −1: the two dots represent the average SI for mixtures that contain the cited compound and do not contain the other; Abscissa = 1: average SI for mixtures that contain both compounds. In this representation, two positive slopes indicate synergism, and two negative slopes denote antagonism. Otherwise, the effects are additive.

The impact of combinations on the selectivity index, here defined as the average IC_50_(MRC_5_ cells)/MIC ratio, was evaluated following the same full factorial experimental design. The effect of factors and interaction plots for antifungal activity are presented in Fig. 3. The two compounds with a significantly positive effect on the selectivity index are (*Z*)-ligustilide (*p* < 0.0001) and eugenol (*p* 0.021). Citral demonstrated no effect, and the other three molecules showed a somewhat negative effect, although it was not statistically significant. (*Z*)-Ligustilide-containing mixtures exhibit an average SI of 0.21 whereas this value is 0.13 in the absence of the compound. The effect is less obvious for eugenol, with an average SI of 0.18 in the presence of the compound, and 0.16 in its absence. The full interaction plot clearly shows a strong positive effect between (*Z*)-ligustilide and eugenol with an average SI of 0.24 for these two compounds in association. Overall, if the presence of eugenol exerts almost no effect on the antifungal potential of (*Z*)-ligustilide, then combinations of these two compounds offer the advantage of decreasing (*Z*)-ligustilide cytotoxicity. On the other hand, (*Z*)-ligustilide/citral combinations, which demonstrated intriguing antimicrobial activity, showed no improved selectivity when compared to (*Z*)-ligustilide alone (average SI of 0.21).

## Discussion

Our goal in this study was to propose an optimized antifungal mixture inspired by plant defensive cocktails. The approach was based on (1) an OPLS analysis followed by (2) a full factorial design taking into account not only the bioactivity of putative antifungal metabolites, but also the interaction effects between these molecules and their potential cellular toxicity.

The first step consisted in the evaluation of the antimicrobial activities of 66 essential oils against *C*. *albicans* and *C*. *parapsilosis* and chemical analysis of EOs composition using GC/MS. (*Z*)- and (*E*)-citral were the major compounds in 6 out of the 13 most active EOs, with total citral proportion ranging from 47.2 to 96.4%. Interestingly, it was recently suggested that large amounts of citral could be responsible for lemon essential oils antifungal effect against *C*. *glabrata*^36^. Eugenol was the major compound of two EOs (*S*. *aromaticum* and *P*. *racemosa*) whereas thymol, (*Z*)-ligustilide, limonene, α-pinene and (−)-citronellol were only identified as major compounds of one of the 13 most active EOs (*Thymus vulgaris* var1, *Levisticum officinale*, *Protium heptaphyllum*, *Sphagneticola trilobata*, and *Pelargonium graveolens* var2). Many of these compounds are described in the literature for anticandidal^27,37,38^ or other antifungal^39,40^ activities.

OPLS analysis allowed for the identification of seven terpenes influential for this model: (*Z*)- and (*E*)-citral, thymol, (*Z*)-ligustilide, eugenol, eugenyl acetate, and (−)-citronellol. Although OPLS analysis did indicate compounds present in high amounts in numerous strongly active EOs such as citral (*Z*/*E* mixture) and eugenol, it also determined additional metabolites such as (−)-citronellol. Moreover, limonene and α-pinene, which were present in very high proportion (90.0 and 77.1%) in active EOs did not exhibit positive regression coefficients because they were also occurring in inactive oils. For example α-pinene was present in variable proportions (from 9 to 55%) of different inactive EOs from the dataset (including *Cinnamomum camphora*, *Cupressus sempervirens*, *Eucalyptus globulus* or *Mikania micrantha*) besides *S*. *trilobata* EO. Limonene was present in proportion ranging from 40 to 96% in *Citrus* EOs, or from 43 to 60% in *Licaria canella* EOs. These results are in accordance with literature data^36^ and with our experimental data, in which limonene and α-pinene tested concurrently were considered inactive pure compounds^41^. Interestingly, Maree *et al*. also identified eugenol as a marker contributing to the antibacterial and anticandidal activity of their selected oils^27^ whereas limonene and α-pinene correlated with samples with poor antimicrobial activity. The statistical tool also discovered thymol and (*Z*)-ligustilide as putative antifungal activity markers, despite the fact that these compounds were only present in one (ligustilide) or two (thymol) of the 66 EOs. These results reinforce the interest of using chemometric approaches to search for active metabolites in complex mixtures.

The second step was devised to understand the interactions between terpenes on a larger scale, rather than focusing on their individual bioactivity and mechanism of action, and eventually design optimal antifungal mixtures. Such an approach may lead to the development of novel effective antimicrobial agents based on the construction of artificial bioinspired blends. The possibilities of using reconstructed EOs, synergistic combinations of terpenes or synergistic combinations of EOs with antifungal drugs have been explored in the literature^22,31,41–44^. While synergy has been found in some cases, our group also found antagonistic effects between terpenes, and noted that the antimicrobial potentials were simply additive in many cases.

With this experimental design approach, it was possible to pinpoint the most active compounds and evaluate synergies and antagonisms altogether. (*Z*)-ligustilide was the strongest antifungal alone and in combination with other terpenes. Our results reinforce the interest in studying this compound in combination with other terpenes to complement existing studies concerning mixtures of (*Z*)-ligustilide and azoles^39,45^, in particular to decrease toxicity rather than attempting to improve antifungal potential. Additionally, a synergistic effect was demonstrated between citral and (*Z*)-ligustilide in combination. Citral was recently reviewed for its effects in laboratory conditions against *Candida* spp., including fluconazole-resistant isolates^37^, and its mechanism of action was investigated^46^. Citral was shown to inhibit *C*. *albicans* growth by affecting membrane integrity and arrest of cell cycle.

However, as for (*Z*)-ligustilide, citral toxicity should be considered in order to better evaluate its potential as antifungal agent. This was exemplified in a recent study considering the genotoxicity of EOs alongside antifungal activity^47^. If (*Z*)-ligustilide and citral were the most active compounds, (*Z*)-ligustilide and eugenol were the two main factors that most contributed to the increase of the SI. A positive interaction effect was recorded between these two compounds. Eugenol was less active, but decreased (*Z*)-ligustilide toxicity without attenuating its antimicrobial activity. These results underscore a potential protective effect of eugenol which will be investigated further with other antifungal compounds, including commercially available drugs.

In conclusion, this work was originally designed as a model study to evaluate the utilization of statistical design for the discovery of innovative antimicrobial natural products and their respective mixtures. To our knowledge, this is the first time that chemometrics and statistical design were associated in a single study to perform a full investigation of putative antimicrobial markers. We highlighted that mixtures of (*Z*)-ligustilide and eugenol could represent a promising starting point for the development of antifungal agents, mostly for topical application. Combinations of these two substances in various ratios should be studied, and it will be enlightening to evaluate the interaction effects of either or both (*Z*)-ligustilide and eugenol with antifungal drugs.

## Methods

### Essential oils

Forty-seven commercially available essential oils were obtained from Aroma-zone (www.aroma-zone.com). Nineteen essential oils were obtained from plants collected and distilled in French Guiana. These plants are not protected and could be collected without restriction at the concerned locations. Collection authorizations were unnecessary at the time of the collection (2006–2007). Plants were collected by Pierre Silland, except for *V*. *americana* wood, obtained from forest exploitation wastes and collected by Alice Rodrigues. Herbarium vouchers were deposited in the French Guiana Herbarium (CAY), where specialists confirmed botanical identification. All collection data are available at: http://publish.plantnet-project.org/project/caypub. The botanical identification of the 66 EOs is available in the supplementary information (Table S1). All names used are accepted botanical names according to botanical databases (www.theplantlist.org; http://www.tropicos.org). The fresh parts collected from each plant were hydrodistilled. All EOs were stored at −18 °C in the absence of light until the subsequent analyses were performed.

### GC-MS analyses

A Shimadzu GC-MSQP2010 fitted with a AOC-20i injector and FID and MS detectors (Shimadzu, Tokyo, Japan) was used for the GC-MS analysis. The GC was run with a nonpolar Supelco MDN-5S fused silica capillary column (30 m × 0.25 mm ID, 0.25 µm film thickness) commonly used for the analysis of VOCs. The injection volume (EO dissolved in chromatography grade hexane) was 1 μL. Helium was used as the carrier gas at a constant flow of 1 mL/min. The column temperature was set at 50 °C for 30 s, then increased from 50–150 °C at 4 °C/min, from 150–175 °C at 1.5 °C/min and from 175–300 °C at 20 °C/min for a total analysis time of 58.42 min. The injector temperature was set to 250 °C and the injection was accomplished with a split ratio of 1/50 throughout the entire run. The MS was operated in the electron impact mode at 70 eV, with a scan range of 40–400 m/z. The temperatures were set to 200 °C for the ion trap, 50 °C for the manifold and 305 °C for the transfer line.

A Varian 450-GC fitted with an MS240 iontrap MS and a Combipal autosampler (Varian Instruments, Sunnyvale, CA, USA) and run with a nonpolar Varian FactorFour VF-5ms column (30 m × 0.25 mm ID, 0.25 μm film) commonly used for the analysis of VOCs was used under the same conditions to assist in compound identification.

### Fungal strains

Bioassays were performed on two clinical isolates of *Candida* sp. (*Candida albicans* LMGO103 and *Candida parapsilosis* LMGO06; Federal University of Goiás Hospital, Brazil), alongside the reference strain *Candida parapsilosis* ATCC22020. Strains were maintained on potato dextrose agar and cultured onto an appropriate new agar plate at 28 °C for 2 days prior to any antimicrobial test.

### Minimal inhibitory concentration (MIC)

The standard microdilution test was used to determine the MIC of the EOs. The experimental details were similar to those described previously^31^ except for yeast suspensions, which were adjusted to 0.5 McFarland standard and then diluted 1:1000 (v/v) with RPMI 1640 medium according to the Clinical and Laboratory Standards Institute^48^. EOs were tested at concentrations ranging from 512 to 1 µg/mL and pure compounds were tested from 64 to 0.125 µg/mL. All assays were conducted in triplicate.

To allow for direct regression of analytical data as a function of antifungal potential, a score representing the global antifungal activity was attributed to each EO. A MIC greater than 512 μg/mL received a 0, a MIC of 512 μg/mL received a 1, a MIC of 256 μg/mL received a 2 and each subsequent reduction in MIC by a factor of 2 increased the number of the score by 1.

### Data pretreatment and chemometric analysis (OPLS model)

The GC/MS profiles recorded on the Shimadzu system were integrated automatically with a slope set to 15,000 and a width of 0.1. The peak corresponding to the solvent stabilizer (di-tert-butyl-2,6-para-cresol, RT = 25.97 min) was removed manually in each analysis. All integrations were checked manually, and some were split, removed or added as necessary. Integrations are reported as a % total integration. All integration tables were gathered in an Excel sheet. The lines were organized by RT. The uncertainty on the RT was estimated at ±0.07 min based on the RT recorded for the solvent stabilizer. A compound code and a putative identification were attributed to each peak. When mass spectra recorded on both systems were considered identical, and when the peaks were not separated by more than 0.15 min, then the same code was assigned to peaks from different EOs. Putative identifications were as described below.

The data were then converted in a pivot table, with compound codes as lines and EOs as columns. The table contained 343 compounds from 66 EOs. Each box contained the relative integration of the compound in each corresponding EO. To reduce the number of variables, the compounds that did not account for at least 9% of one EO were removed. Sixty compounds remained. An orthogonal partial least square analysis (OPLS) was performed with Umetrics Simca 15. X-variables were the compound relative proportions in each EO, and the Y-variable was the note of antifungal activity recorded for each EO. One orthogonal component was sufficient to capture most of the inertia of the model. R^2^Y (cum) was 67%, R^2^X (cum) 4%, and Q^2^ (cum) 34%. The permutations plot provided in supporting information (Fig. S1) suggests that the model is valid. The regression coefficients for variables with positive coefficients are reported in Fig. 1. A full data table is provided in the Supporting Information (Table S2). Due to controversies over SIMCA permutation plots^49^ the model was concurrently assessed using R *ropls* package (https://www.bioconductor.org/packages/devel/bioc/vignettes/ropls/inst/doc/ropls-vignette.html). In this analysis R^2^Y(cum) was 79%, R^2^X(cum) 10%, and Q^2^(cum) 33%. This permutations plot is provided in supporting information (Fig. S2) alongside with inertia barplot, observation diagnostics of the model and scores plot. Loadings plot obtained using Umetrics Simca 15 is presented as Fig. S3.

### Identification of the putatively active compounds

The 12 most active compounds as defined by OPLS analysis were identified by (i) GC retention indices (RI), (ii) computer matching with commercial mass spectral libraries (NIST 98 MS, ADAMS)^50^, (iii) comparisons of RI and spectra with those from previous work^31,51^ and an in-house library of analyses of commercial EOs of known composition (Aroma-Zone), and (iv) confirmation by comparison with identifications provided in the literature.

### Design of experiment

A full factorial experimental design including the six putative antifungal markers citral (Ci), thymol (Th), (*Z*)-ligustilide (Li), eugenol (Eu), eugenyl acetate (EA), and (−)-citronellol (–C) was constructed with Minitab 16 and XLStat 2014. All 64 possible combinations were randomly tested (order defined by the software) in duplicate against *Candida* spp. for antifungal activity, and MRC_5_ cells for cytotoxicity. All compounds were distributed equally in the tested mixtures. All combinations were tested at concentrations ranging from 512 to 1 µg/mL such as performed for crude EOs.

The effect of factors and interaction plots are displayed in Figs 2 and 3 for antifungal activity and antifungal selectivity, respectively. The plots were obtained using Minitab 16 data and drawn with Graphpad Prism 5 software. A full data table is provided in Supporting Information (Table S3).

For antifungal activity, R^2^ was 57%, R^2^ (adj) 53%, and R^2^ (pred) 54%. The analysis of variance of the model was a follows in Table 2.

**Table 2:** Analysis of variance for the antifungal activity model. Computed against model Y= 0.

Source	Degrees of Freedom	Sum of squares	Mean squares	F	Pr > F
Model	6	130.719	**21.786**	**12.621**	**<0.0001**
Error	57	98.391	**1.726**		
Corrected Total	63	229.109

**Table 3:** Analysis of variance for the antifungal selectivity model.

Source	Degrees of Freedom	Sum of squares	Mean squares	F	Pr > F
Model	6	0.215	0.036	7.930	<0.0001
Error	57	0.258	0.005		
Corrected Total	63	0.473			

Computed against model Y = 0.

For antifungal selectivity, R^2^ was 46%, R^2^(adj) 40%, and R^2^(pred) 41%. The analysis of variance of the model was a follows in Table 3.

### Cytotoxicity assays

Cytotoxicity assays were conducted with MRC5 (normal lung tissue of a 14-week-old male fetus) cell lines using the procedure described by Tempête *et al*.^52^; docetaxel was used as positive control.

## Supplementary information

Supporting information

## Data Availability

All data generated or analyzed during this study are included in this published article (and its Supporting Information files).

## References

[1] Woodhouse M, Farrar J (2014). Policy: an intergovernmental panel on antimicrobial resistance. Nature.

[2] Reardon S (2014). Antibiotic resistance sweeping developing world. Nature.

[3] Shor E, Perlin DS (2015). Coping with stress and the emergence of multidrug resistance in fungi. PLoS Pathog.

[4] Paiva JA (2013). Adding risk factors for potentially resistant pathogens, increasing antibiotic pressure and risk creating the ‘untreatable bacteria’: time to change direction. Intensive Care Med.

[5] Kåhrström CT (2013). Entering a post-antibiotic era?. Nat. Rev. Microbiol..

[6] World Health Organization Antimicrobial Resistance: Global Report on Surveillance 2014 (WHO, 2014).

[7] D’Costa VM (2011). Antibiotic resistance is ancient. Nature.

[8] Bhullar K (2012). Antibiotic resistance is prevalent in an isolated cave microbiome. PLoS One.

[9] Bollenbach T (2015). Antimicrobial interactions: mechanisms and implications for drug discovery and resistance evolution. Curr. Opin. Microbiol..

[10] Worthington RJ, Melander C (2013). Combination approaches to combat multi-drug resistant bacteria. Trends Biotechnol..

[11] Brunetti C, Guidi L, Sebastiani F, Tattini M (2015). Isoprenoids and phenylpropanoids are key components of the antioxidant defense system of plants facing severe excess light stress. Environ. Exp. Bot..

[12] Gershenzon J, Dudareva N (2007). The function of terpene natural products in the natural world. Nat. Chem. Biol..

[13] Richards LA (2016). Phytochemical diversity and synergistic effects on herbivores. Phytochem. Rev..

[14] Jones AC, Blum JE, Pawlik JR (2005). Testing for defensive synergy in Caribbean sponges: bad taste or glass spicules?. J. Exp. Mar. Biol. Ecol..

[15] Mason PA, Singer MS (2015). Defensive mixology: combining acquired chemicals towards defence. Funct. Ecol..

[16] Unsicker SB, Kunert G, Gershenzon J (2009). Protective perfumes: the role of vegetative volatiles in plant defense against herbivores. Curr. Opin. Plant Biol..

[17] Baby, S. & George, V. Essential oils and new antimicrobial strategies in *New strategies combating bacterial infection* (ed. Ahmad, I. & Aqil, F.) 165–203 (Wiley, 2009).

[18] Lopes G, Pinto E, Salgueiro L (2017). Natural products: an alternative to conventional therapy for dermatophytosis?. Mycopathologia.

[19] Yap PSX, Yiap BC, Ping HC, Lim SHE (2014). Essential oils, a new horizon in combating bacterial antibiotic resistance. Open Microbiol. J..

[20] Tian J (2012). The mechanism of antifungal action of essential oil from dill (*Anethum graveolens* L.) on *Aspergillus flavus*. PLoS One.

[21] Tserennadmid R (2011). Anti-yeast activities of some essential oils in growth medium, fruit juices and milk. Int. J. Food Microbiol..

[22] Khoury M (2014). Chemical composition and antimicrobial activity of the essential oil of *Juniperus excelsa* M. Bieb. growing wild in Lebanon. Chem. Biodivers..

[23] Fairlamb AH, Gow NAR, Matthews KR, Waters AP (2016). Drug resistance in eukaryotic microorganisms. Nat. Microbiol..

[24] Harvey AL, Edrada-Ebel R, Quinn RJ (2015). The re-emergence of natural products for drug discovery in the genomics era. Nat. Rev. Drug Discov..

[25] Ali K (2012). NMR spectroscopy and chemometrics as a tool for anti-TNFα activity screening in crude extracts of grapes and other berries. Metabolomics.

[26] Rolli E, Marieschi M, Maietti S, Sacchetti G, Bruni R (2014). Comparative phytotoxicity of 25 essential oils on pre- and post-emergence development of *Solanum lycopersicum* L.: A multivariate approach. Ind. Crops Prod..

[27] Maree J, Kamatou G, Gibbons S, Viljoen A, Van Vuuren S (2014). The application of GC-MS combined with chemometrics for the identification of antimicrobial compounds from selected commercial essential oils. Chemometr. Intell. Lab. Syst..

[28] Blainski A (2017). Antibacterial activity of *Limonium brasiliense* (Baicuru) against multidrug-resistant bacteria using a statistical mixture design. J. Ethnopharmacol..

[29] Butassi E (2015). Synergistic mutual potentiation of antifungal activity of *Zuccagnia punctata* Cav. and *Larrea nitida* Cav. extracts in clinical isolates of *Candida albicans* and *Candida glabrata*. Phytomedicine.

[30] Tak JH, Isman MB (2017). Enhanced cuticular penetration as the mechanism of synergy for the major constituents of thyme essential oil in the cabbage looper, *Trichoplusia* ni. Ind. Crops Prod..

[31] Houël E (2014). *In vitro* antidermatophytic activity of *Otacanthus azureus* (Linden) Ronse essential oil alone and in combination with azoles. J. Appl. Microbiol..

[32] Cos P, Vlietinck AJ, Berghe DV, Maes L (2006). Anti-infective potential of natural products: How to develop a stronger *in vitro* “proof-of-concept”. J. Ethnopharmacol..

[33] Gertsch J (2009). How scientific is the science in ethnopharmacology? Historical perspectives and epistemological problems. J. Ethnopharmacol..

[34] Shang X, Zhong X, Tian X (2016). Metabolomics of papillary thyroid carcinoma tissues. Potential biomarkers for diagnosis and promising targets for therapy. Tumor Biol..

[35] Wheelock AM, Wheelock CE (2013). Trials and tribulations of ‘omics data analysis: assessing quality of SIMCA-based multivariate models using examples from pulmonary medicine. Mol. Biosyst..

[36] Białoń M, Krzyśko-Łupicka T, Koszałkowska M, Wieczorek PP (2014). The influence of chemical composition of commercial lemon essential oils on the growth of *Candida* strains. Mycopathologia.

[37] Lu M, Li T, Wan J, Li X, Sun S (2017). Antifungal effects of phytocompounds on *Candida* species alone and in combination with fluconazole. Int. J. Antimicrob. Agents.

[38] Musthafa KS, Hmoteh J, Thamjarungwong B, Voravuthikunchai SP (2016). Antifungal potential of eugenyl acetate against clinical isolates of *Candida* species. Microb. Pathog..

[39] Sim Y, Shim S (2008). Combinatorial anti-*Trichophyton* effects of *Ligusticum chuanxiong* essential oil components with antibiotics. Arch. Pharm. Res..

[40] Pannek J, Gach J, Boratynski F, Olejniczak T (2018). Antimicrobial activity of extracts and phthalides occurring in Apiaceae plants. Phytother. Res..

[41] Houël E (2015). Therapeutic switching: from antidermatophytic essential oils to new leishmanicidal products. Mem. Inst. Oswaldo Cruz.

[42] Fahed L (2017). Essential oils composition and antimicrobial activity of six conifers harvested in Lebanon. Chem. Biodivers..

[43] Mulyaningsih S, Sporer F, Zimmermann S, Reichling J, Wink M (2010). Synergistic properties of the terpenoids aromadendrene and 1,8-cineole from the essential oil of *Eucalyptus globulus* against antibiotic-susceptible and antibiotic-resistant pathogens. Phytomedicine.

[44] Van Vuuren SF, Viljoen AM (2007). Antimicrobial activity of limonene enantiomers and 1,8-cineole alone and in combination. Flavour Fragr. J..

[45] Sim Y, Shin S (2010). Anti-*Aspergillus* activities of the *Ligusticum chuanxiong* essential oil alone and in combination with antibiotics. Nat. Prod. Sci..

[46] Zore GB, Thakre AD, Jadhav S, Karuppayil SM (2011). Terpenoids inhibit *Candida albicans* growth by affecting membrane integrity and arrest of cell cycle. Phytomedicine.

[47] Ruiz-Pérez NJ (2016). Antimycotic activity and genotoxic evaluation of *Citrus sinensis* and *Citrus latifolia* essential oils. Sci. Rep..

[48] CLSI (Clinical and Laboratory Standards Institute). Reference method for broth dilution antifungal susceptibility testing of yeasts; Approved Standard, 2nd edn. Document M27-A3 (2008).

[49] Triba MN (2015). PLS/OPLS models in metabolomics: the impact of permutation of dataset rows on the K-fold cross-validation quality parameters. Mol. BioSyst..

[50] Adams, R.P. In *Identification of essential oil components by gas chromatography/mass spectrometry*, *4th ed*. (Allured Publishing Corporation, 2007).

[51] Courtois EA (2009). Diversity of the volatile organic compounds emitted by 55 species of tropical trees: a survey in French Guiana. J. Chem. Ecol..

[52] Tempête C, Werner G, Favre F, Roja A, Langlois N (1995). *In vitro* cytostatic activity of 9-demethoxyporothramcyn B. Eur. J. Med. Chem..

