# Supplementary material for: The antifungal potential of (Z)-ligustilide and the protective effect of eugenol demonstrated by a chemometric approach
Source: Sci Rep. 2019 Jun 19;9:8729. doi: 10.1038/s41598-019-45222-y (PMC6584663; doi:10.1038/s41598-019-45222-y)
Supplement: Supplementary file 1 — Supporting information [file 41598_2019_45222_MOESM1_ESM.docx]

**Supporting** **information**

**The antifungal potential of (*Z*)-ligustilide and the protective effect of eugenol demonstrated by a chemometric approach**

Alice M. S. Rodrigues^1^, Véronique Eparvier^2^, Guillaume Odonne^3^, Nadine Amusant^4^, Didier Stien^1,*^, Emeline Houël^5,*^

^1^Sorbonne Université, CNRS, Laboratoire de Biodiversité et Biotechnologie Microbienne, LBBM, Observatoire Océanologique, 66650 Banyuls-sur-mer, France

^2^CNRS, Institut de Chimie des Substances Naturelles, UPR2301, Université Paris-Saclay, 91198 Gif-sur-Yvette, France

^3^Laboratoire Ecologie, Evolution, Interactions des Systèmes Amazoniens (LEEISA), CNRS, Université de Guyane, IFREMER, 97300 Cayenne, France

^4^CIRAD, UMR EcoFoG, AgroParisTech, CNRS, INRA, Université des Antilles, Université de Guyane, 97300 Cayenne, France

^5^CNRS, UMR EcoFoG, AgroParisTech, Cirad, INRA, Université des Antilles, Université de Guyane, 97300 Cayenne, France

*didier.stien@cnrs.fr; emeline.houel@ecofog.gf

**Table S1** Botanical identification, anticandidal activity (minimum inhibitory concentrations (MIC), µg/mL) and antifungal activity score (AAS) measured for the selected essential oils. The antifungal activity score is calculated based on the measured minimum inhibitory concentrations (MICs), according to the following scheme: > 512 µg/mL = 0; 512 µg/mL = 1; 256 = 2; 128 = 3; 64 = 4. The final score is obtained by adding together the values obtained for each fungal strain.

**Table S2** Full data table of essential oil bioactivity chemometric analysis by OPLS

**Figure S1.** Permutations plot (100 permutations) displaying R2-values (green circles) and Q2-values (blue squares). The solid line represents the R2 regression line and the dashed line represents the Q2 regression line.

**Figure S2**. OPLS summary plot obtained using R *ropls* package. Top left: significance diagnostic using permutation plots displaying R^2^-values (light grey) and Q^2^-values (dark grey). The solid line represents the R^2^ and Q^2^ regression lines. Top right: inertia barplot. The graphic indicates that 1 orthogonal component may be sufficient to capture most of the inertia. Bottom left: observation diagnostic. Bottom right: scores plot. The number of components and the cumulative R^2^X, R^2^Y and Q^2^Y are indicated below the plot.

**Figure S3**. OPLS loading scatter plot obtained using SIMCA 15.

**Table S3.** Full factorial design and results obtained for antifungal and cytotoxic activities. The anticandidal activity (minimum inhibitory concentrations (MIC), µg/mL) and cytotoxicity (IC50, µg/mL) are indicated for each combination. The antifungal activity score is calculated based on the measured minimum inhibitory concentrations (MICs), according to the following scheme: > 512 µg/mL = 0; 512 µg/mL = 1; 256 = 2; 128 = 3; 64 = 4. The final score is obtained by adding together the values obtained for each fungal strain.

**Table S1** Botanical identification, anticandidal activity (minimum inhibitory concentrations (MIC), µg/mL) and antifungal activity score (AAS) measured for the selected essential oils. The antifungal activity score is calculated based on the measured minimum inhibitory concentrations (MICs), according to the following scheme: > 512 µg/mL = 0; 512 µg/mL = 1; 256 = 2; 128 = 3; 64 = 4. The final score is obtained by adding the values obtained for each fungal strain.

| **Botanical identification, origin* and herbarium voucher number**** | **MIC *C.a.* LMGO 103***** | **MIC *C.p.* ATCC 22020***** | **MIC *C.p.* LMGO 06***** | **Antifungal Activity Score** |
| --- | --- | --- | --- | --- |
| *Thymus vulgaris* var1 (c) | 64 | 64 | 64 | 12 |
| *Levisticum officinale* (c) | 64 | 64 | 128 | 11 |
| *Cymbopogon citratus* (c) | 128 | 128 | 64 | 10 |
| *Protium heptaphyllum* (l, PS20) | 128 | 64 | 128 | 10 |
| *Cymbopogon citratus* (l, sample 1, PS40) | 128 | 256 | 64 | 9 |
| *Cymbopogon citratus* (l, sample 2, PS40) | 256 | 128 | 128 | 8 |
| *Syzygium aromaticum* (c,) | 256 | 128 | 128 | 8 |
| *Backhousia citriodora* (c) | 256 | 256 | 128 | 7 |
| *Leptospermum petersonii* (c) | 256 | >512 | 128 | 5 |
| *Melissa officinalis* (c) | 256 | 512 | 256 | 5 |
| *Pelargonium graveolens* var2 (c) | 512 | 256 | 512 | 5 |
| *Pimenta racemosa* (c) | 256 | 256 | 512 | 5 |
| *Sphagneticola trilobata* (l, PS4)) | 512 | 256 | 256 | 5 |
| *Pelargonium graveolens* var1 (c) | 512 | 256 | 512 | 4 |
| *Pimenta racemosa* (l, PS26) | 512 | 512 | 256 | 4 |
| *Cryptocarya agathophylla* var2 (c) | 512 | 256 | 512 | 4 |
| *Cymbopogon winterianus* (c) | >512 | 256 | 512 | 3 |
| *Mentha x piperita* var1(c) | 512 | 512 | 512 | 3 |
| *Daucus carota* (c) | >512 | 512 | 512 | 2 |
| *Thymus mastichina* (c) | >512 | 512 | 512 | 2 |
| *Cedrus atlantica* (c) | >512 | 512 | >512 | 1 |
| *Citrus clementina* (c) | >512 | 512 | >512 | 1 |
| *Eucalyptus dives* (c) | >512 | >512 | 512 | 1 |
| *Lavandula angustifolia* (c) | >512 | >512 | 512 | 1 |
| *Mentha arvensis* (c) | 512 | >512 | >512 | 1 |
| *Thymus vulgaris* var2 (c) | 512 | >512 | >512 | 1 |
| *Piper aduncum* (l, PS22) | >512 | 512 | >512 | 1 |
| *Unxia camphorata* (l, PS7) | 512 | >512 | >512 | 1 |
| *Cinnamomum camphora* (c) | >512 | >512 | >512 | 0 |
| *Cinnamomum tamala* (c) | >512 | >512 | >512 | 0 |
| *Citrus aurantiifolia* (c) | >512 | >512 | >512 | 0 |
| *Citrus aurantium* var1 (c) | >512 | >512 | >512 | 0 |
| *Citrus aurantium* var2 (c) | >512 | >512 | >512 | 0 |
| *Citrus reticulata* var1 (c) | >512 | >512 | >512 | 0 |
| *Citrus reticulata* var2 (c) | >512 | >512 | >512 | 0 |
| *Citrus sinensis* (c) | >512 | >512 | >512 | 0 |
| *Citrus x bergamia* (c) | >512 | >512 | >512 | 0 |
| *Citrus x limonum* (c) | >512 | >512 | >512 | 0 |
| *Copaifera officinalis* (c) | >512 | >512 | >512 | 0 |
| *Corymbia citriodora* (c) | >512 | >512 | >512 | 0 |
| *Croton nuntians* (l, PS11) | >512 | >512 | >512 | 0 |
| *Cupressus sempervirens* (c) | >512 | >512 | >512 | 0 |
| *Eucalyptus globulus* (c) | >512 | >512 | >512 | 0 |
| *Eucalyptus polybractea* (c) | >512 | >512 | >512 | 0 |
| *Eucalyptus radiata* (c) | >512 | >512 | >512 | 0 |
| *Eucalyptus smithii* (c) | >512 | >512 | >512 | 0 |
| *Lavandula hybrida* (c) | >512 | >512 | >512 | 0 |
| *Lavandula latifolia* (c) | >512 | >512 | >512 | 0 |
| *Lavandula x intermedia* (c) | >512 | >512 | >512 | 0 |
| *Licaria canella* (l, fresh bark, PS16) | >512 | >512 | >512 | 0 |
| *Licaria canella* (l, wood, PS16) | >512 | >512 | >512 | 0 |
| *Licaria cannella* (l, dry bark, PS16) | >512 | >512 | >512 | 0 |
| *Melaleuca ericifolia* (c) | >512 | >512 | >512 | 0 |
| *Mentha x piperita* var2 (c) | >512 | >512 | >512 | 0 |
| *Mikania micrantha* (l, PS12) | >512 | >512 | >512 | 0 |
| *Pinus caribaea* (l, PS14) | >512 | >512 | >512 | 0 |
| *Piper hispidum* (l, sample 1, PS23) | >512 | >512 | >512 | 0 |
| *Piper hispidum* (l, sample 2, PS23) | >512 | >512 | >512 | 0 |
| *Piper marginatum* (l, PS21) | >512 | >512 | >512 | 0 |
| *Cryptocarya agathophylla* (c) | >512 | >512 | >512 | 0 |
| *Rosmarinus officinalis* (c) | >512 | >512 | >512 | 0 |
| *Thymus vulgaris* var3 (c) | >512 | >512 | >512 | 0 |
| *Turnera odorata* (l, PS38) | >512 | >512 | >512 | 0 |
| *Varronia curassavica* (l, PS5) | >512 | >512 | >512 | 0 |
| *Vouacapoua americana* (l, AR6) | >512 | >512 | >512 | 0 |
| *Zingiber officinale* (c) | >512 | >512 | >512 | 0 |
| * (c): commercial; (l): laboratory |  |  |  |  |
| ** for the plants collected in French Guiana; plant collectors: Pierre Silland (PS) and Alice Rodrigues (AR) | | |  |  |
| *** *C.a. Candida albicans; C.p. Candida parapsilosis* | |  |  |  |

**Table S2** Full data table of essential oil bioactivity chemometric analysis by OPLS

| **Var ID (Primary)** | **M1.CoeffCS[1] (Anticandidal activity)** | **2.44693 * M1.CoeffCS[1] (Anticandidal activity)cvSE** |
| --- | --- | --- |
| FU | 0.226922 | 0.133096 |
| FF | 0.222602 | 0.120642 |
| GI | 0.160261 | 0.370596 |
| PS | 0.14514 | 0.338895 |
| HL | 0.109145 | 0.159558 |
| MH | 0.0969221 | 0.227263 |
| AT | 0.0769188 | 0.149939 |
| FA | 0.0655938 | 0.0892951 |
| FV | 0.0590428 | 0.0963044 |
| JN | 0.0496585 | 0.111952 |
| FO | 0.0407129 | 0.0893108 |
| EL | 0.0323164 | 0.0784412 |
| DR | 0.0280096 | 0.0659178 |
| DG | 0.0072838 | 0.0124753 |
| BC | 0.00566723 | 0.153568 |
| OK | 0.000487046 | 0.00437213 |
| EE | -0.0000164412 | 0.00648229 |
| IB | -0.00212655 | 0.053949 |
| DF | -0.00324156 | 0.076832 |
| FP | -0.00596497 | 0.0471081 |
| AI | -0.0147207 | 0.101058 |
| KA | -0.0155855 | 0.0338889 |
| KU | -0.0155855 | 0.0338889 |
| LV | -0.0155855 | 0.0338889 |
| OO | -0.0168079 | 0.0393681 |
| BK | -0.018436 | 0.136655 |
| DB | -0.0212132 | 0.079684 |
| MC | -0.0297421 | 0.0723836 |
| BE | -0.0298598 | 0.076742 |
| MN | -0.0316515 | 0.0763212 |
| EQ | -0.031658 | 0.0725101 |
| GD | -0.031658 | 0.0712443 |
| HV | -0.031658 | 0.0712443 |
| KE | -0.031658 | 0.0712443 |
| LO | -0.031658 | 0.0762484 |
| OB | -0.031658 | 0.0762484 |
| RF | -0.031658 | 0.0748603 |
| BL | -0.032161 | 0.0744401 |
| OW | -0.033809 | 0.0743698 |
| GB | -0.0380244 | 0.0694962 |
| NX | -0.0381101 | 0.069763 |
| AF | -0.0391985 | 0.0691309 |
| KW | -0.0399454 | 0.0744402 |
| AY | -0.0404889 | 0.0319635 |
| LL | -0.0451194 | 0.0725257 |
| JJ | -0.0461541 | 0.0573595 |
| LR | -0.0528982 | 0.0679319 |
| BD | -0.0560966 | 0.124701 |
| LF | -0.0593095 | 0.0641676 |
| AN | -0.0604593 | 0.0745833 |
| NN | -0.0605584 | 0.0657862 |
| IA | -0.0632474 | 0.0686619 |
| DA | -0.0640168 | 0.0646835 |
| AP | -0.0657449 | 0.102099 |
| IV | -0.0700415 | 0.0753341 |
| KD | -0.0701151 | 0.0678144 |
| BF | -0.0746817 | 0.0687898 |
| FM | -0.0762967 | 0.0784396 |
| EF | -0.0767521 | 0.0873149 |
| CE | -0.0881575 | 0.0646603 |


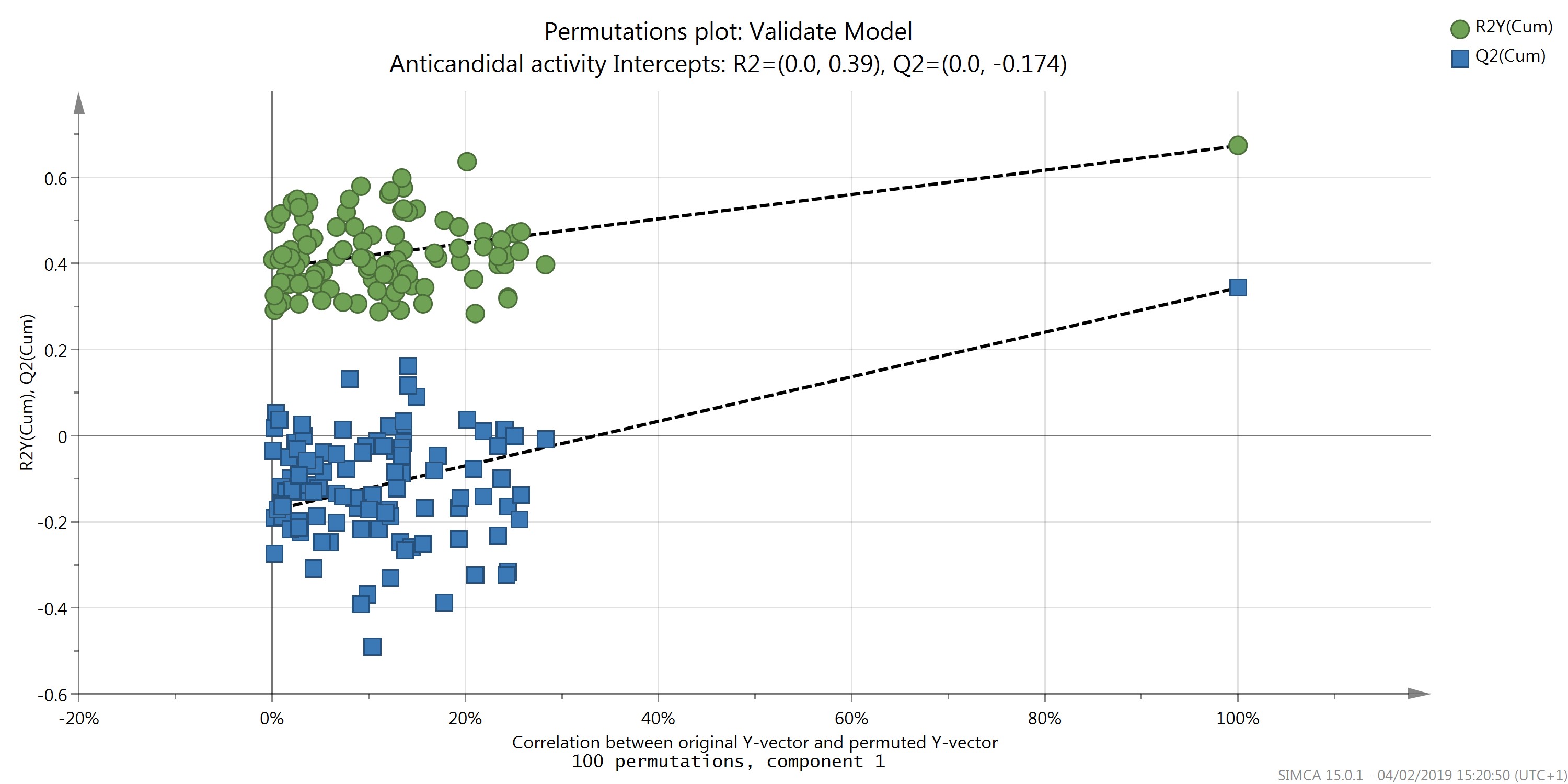


**Figure S1.** Permutations plot (100 permutations) obtained using SIMCA software and displaying R^2^-values (green circles) and Q^2^-values (blue squares). The solid line represents the R^2^ regression line and the dashed line represents the Q^2^ regression line.


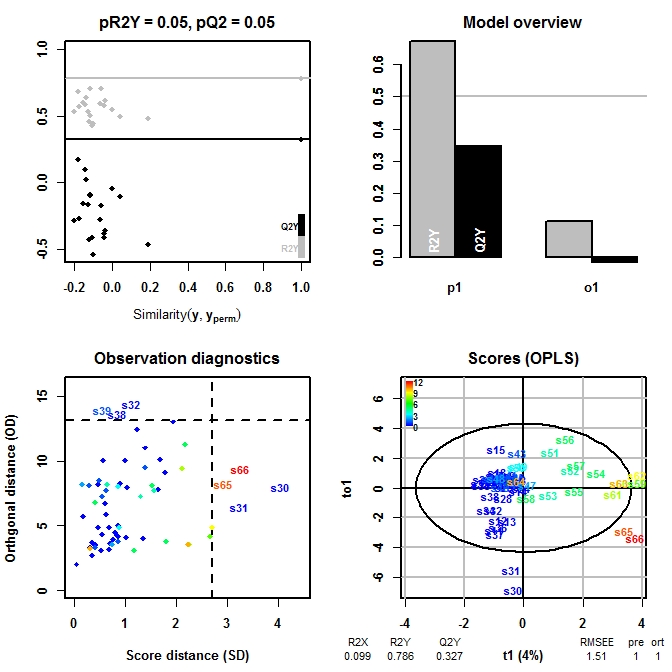


**Figure S2**. OPLS summary plot obtained using R *ropls* package. Top left: significance diagnostic using permutation plots displaying R^2^-values (light grey) and Q^2^-values (dark grey). The solid line represents the R^2^ and Q^2^ regression lines. Top right: inertia barplot. The graphic indicates that 1 orthogonal component may be sufficient to capture most of the inertia. Bottom left: observation diagnostic. Bottom right: scores plot. The number of components and the cumulative R^2^X, R^2^Y and Q^2^Y are indicated below the plot.

**
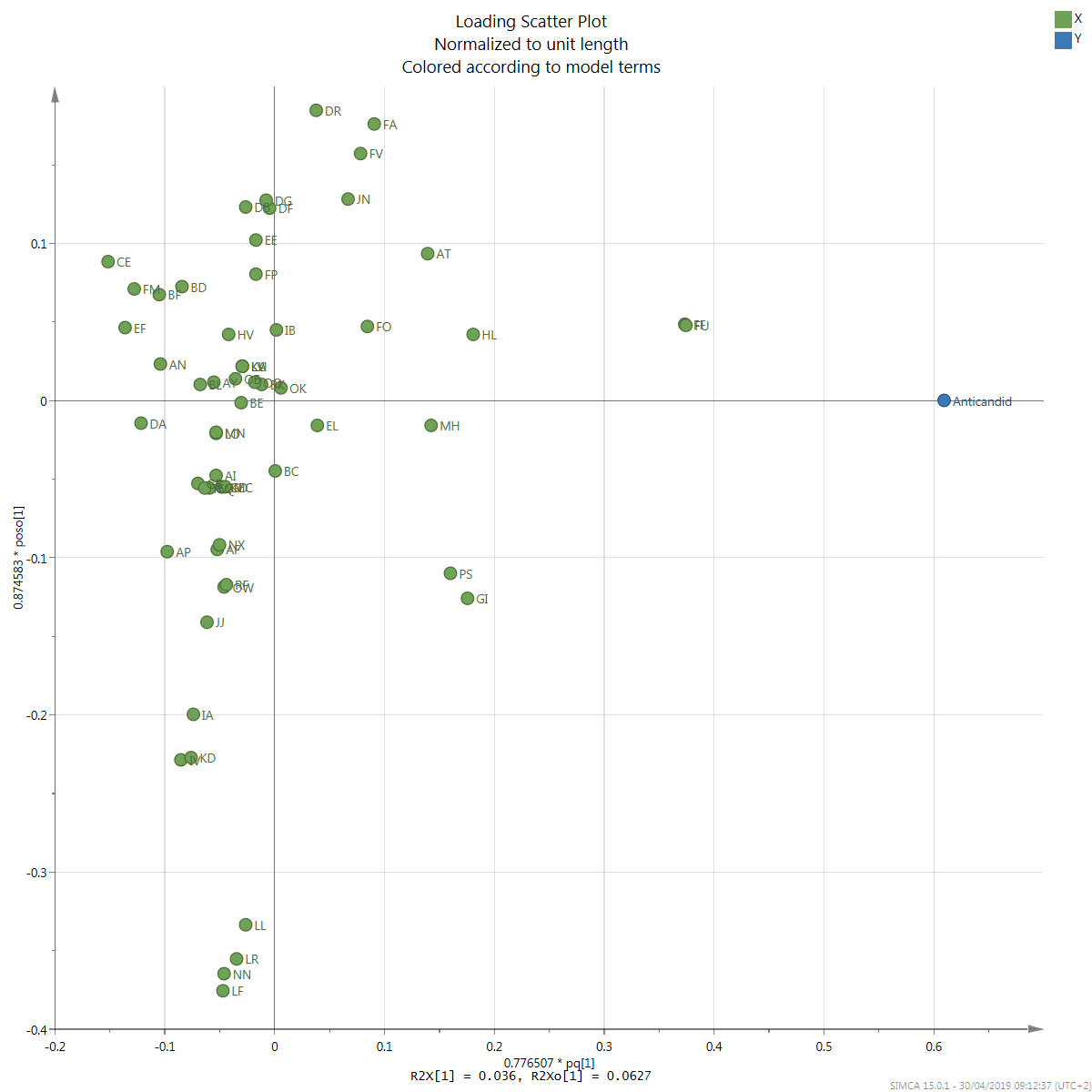
**

**Figure S3**. OPLS loadings plot obtained using SIMCA 15. Letters indicate compounds from the analysis. Points for compounds FF and FU are superimposed. FF: (*Z*)-Citral; FU: (*E*)-Citral; GI: Thymol; PS: (*Z*)-Ligustilide; HL: Eugenol; MH: Eugenyl acetate; AT: β-Pinene; FA: (–)-Citronellol; FV: (–)-Citronellyl formate; JN: Aristolene; FO: (*E*)-Anethole; EL: Estragol.

**Table S3.** Full factorial design and results obtained for antifungal and cytotoxic activities. The anticandidal activity (minimum inhibitory concentrations (MIC), µg/mL) and cytotoxicity (IC50, µg/mL) are indicated for each combination. The antifungal activity score is calculated based on the measured minimum inhibitory concentrations (MICs), according to the following scheme: > 512 µg/mL = 0; 512 µg/mL = 1; 256 = 2; 128 = 3; 64 = 4. The final score is obtained by adding the values obtained for each fungal strain.

| **StdOrder** | **RunOrder** | **Ligustilide** | **Eugenol** | **Citral** | **Thymol** | **Eugenyl acetate** | **β-Citronellol** | **MIC *C.a.* ATCC10231** | **MIC *C.p.* ATCC22019** | **Antifungal activity score** | **IC_50_ MRC5** | **Average SI** |
| --- | --- | --- | --- | --- | --- | --- | --- | --- | --- | --- | --- | --- |
| 1 | 16 | -1 | -1 | -1 | -1 | -1 | -1 | > 512 | > 512 | 0 | - | - |
| 2 | 5 | 1 | -1 | -1 | -1 | -1 | -1 | 32 | 64 | 9 | 4.5 | 0.09 |
| 3 | 22 | -1 | 1 | -1 | -1 | -1 | -1 | 512 | 256 | 3 | 45.0 | 0.12 |
| 4 | 18 | 1 | 1 | -1 | -1 | -1 | -1 | 16 | 32 | 11 | 13.5 | 0.56 |
| 5 | 2 | -1 | -1 | 1 | -1 | -1 | -1 | 64 | 64 | 8 | 8.0 | 0.12 |
| 6 | 57 | 1 | -1 | 1 | -1 | -1 | -1 | 32 | 32 | 10 | 6.0 | 0.19 |
| 7 | 29 | -1 | 1 | 1 | -1 | -1 | -1 | 64 | 64 | 8 | 13.0 | 0.20 |
| 8 | 1 | 1 | 1 | 1 | -1 | -1 | -1 | 32 | 32 | 10 | 10.5 | 0.33 |
| 9 | 25 | -1 | -1 | -1 | 1 | -1 | -1 | 128 | 64 | 7 | 9.0 | 0.09 |
| 10 | 9 | 1 | -1 | -1 | 1 | -1 | -1 | 64 | 64 | 8 | 6.5 | 0.10 |
| 11 | 64 | -1 | 1 | -1 | 1 | -1 | -1 | 128 | 128 | 6 | 13.0 | 0.10 |
| 12 | 59 | 1 | 1 | -1 | 1 | -1 | -1 | 64 | 64 | 8 | 11.0 | 0.17 |
| 13 | 26 | -1 | -1 | 1 | 1 | -1 | -1 | 64 | 64 | 8 | 9.5 | 0.15 |
| 14 | 30 | 1 | -1 | 1 | 1 | -1 | -1 | 64 | 64 | 8 | 10.5 | 0.16 |
| 15 | 4 | -1 | 1 | 1 | 1 | -1 | -1 | 64 | 64 | 8 | 12.0 | 0.19 |
| 16 | 10 | 1 | 1 | 1 | 1 | -1 | -1 | 64 | 64 | 8 | 13.5 | 0.21 |
| 17 | 27 | -1 | -1 | -1 | -1 | 1 | -1 | 512 | 128 | 4 | 47.5 | 0.15 |
| 18 | 46 | 1 | -1 | -1 | -1 | 1 | -1 | 64 | 64 | 8 | 11.5 | 0.18 |
| 19 | 52 | -1 | 1 | -1 | -1 | 1 | -1 | 256 | 128 | 5 | 37.5 | 0.19 |
| 20 | 39 | 1 | 1 | -1 | -1 | 1 | -1 | 64 | 64 | 8 | 20.0 | 0.31 |
| 21 | 56 | -1 | -1 | 1 | -1 | 1 | -1 | 512 | 128 | 4 | 13.0 | 0.04 |
| 22 | 32 | 1 | -1 | 1 | -1 | 1 | -1 | 64 | 64 | 8 | 10.5 | 0.16 |
| 23 | 8 | -1 | 1 | 1 | -1 | 1 | -1 | 128 | 128 | 6 | 15.5 | 0.12 |
| 24 | 24 | 1 | 1 | 1 | -1 | 1 | -1 | 64 | 64 | 8 | 13.5 | 0.21 |
| 25 | 12 | -1 | -1 | -1 | 1 | 1 | -1 | 128 | 128 | 6 | 10.5 | 0.08 |
| 26 | 58 | 1 | -1 | -1 | 1 | 1 | -1 | 64 | 64 | 8 | 12.5 | 0.19 |
| 27 | 13 | -1 | 1 | -1 | 1 | 1 | -1 | 256 | 128 | 5 | 17.5 | 0.09 |
| 28 | 14 | 1 | 1 | -1 | 1 | 1 | -1 | 128 | 64 | 7 | 16.5 | 0.17 |
| 29 | 7 | -1 | -1 | 1 | 1 | 1 | -1 | 128 | 128 | 6 | 11.5 | 0.09 |
| 30 | 20 | 1 | -1 | 1 | 1 | 1 | -1 | 64 | 64 | 8 | 11.5 | 0.18 |
| 31 | 40 | -1 | 1 | 1 | 1 | 1 | -1 | 128 | 128 | 6 | 11.0 | 0.08 |
| 32 | 54 | 1 | 1 | 1 | 1 | 1 | -1 | 64 | 64 | 8 | 13.5 | 0.21 |
| 33 | 63 | -1 | -1 | -1 | -1 | -1 | 1 | 256 | 256 | 4 | 8.0 | 0.03 |
| 34 | 38 | 1 | -1 | -1 | -1 | -1 | 1 | 64 | 32 | 9 | 8.5 | 0.18 |
| 35 | 3 | -1 | 1 | -1 | -1 | -1 | 1 | 512 | 128 | 4 | 18.5 | 0.06 |
| 36 | 43 | 1 | 1 | -1 | -1 | -1 | 1 | 64 | 64 | 8 | 14.5 | 0.23 |
| 37 | 41 | -1 | -1 | 1 | -1 | -1 | 1 | 128 | 128 | 6 | 10.5 | 0.08 |
| 38 | 49 | 1 | -1 | 1 | -1 | -1 | 1 | 64 | 32 | 9 | 9.5 | 0.20 |
| 39 | 11 | -1 | 1 | 1 | -1 | -1 | 1 | 256 | 128 | 5 | 15.0 | 0.08 |
| 40 | 17 | 1 | 1 | 1 | -1 | -1 | 1 | 64 | 64 | 8 | 15.5 | 0.24 |
| 41 | 48 | -1 | -1 | -1 | 1 | -1 | 1 | 128 | 64 | 7 | 11.5 | 0.12 |
| 42 | 42 | 1 | -1 | -1 | 1 | -1 | 1 | 64 | 64 | 8 | 9.0 | 0.14 |
| 43 | 35 | -1 | 1 | -1 | 1 | -1 | 1 | 256 | 128 | 5 | 15.5 | 0.08 |
| 44 | 44 | 1 | 1 | -1 | 1 | -1 | 1 | 128 | 64 | 7 | 13.5 | 0.14 |
| 45 | 6 | -1 | -1 | 1 | 1 | -1 | 1 | 256 | 128 | 5 | 8.5 | 0.04 |
| 46 | 15 | 1 | -1 | 1 | 1 | -1 | 1 | 64 | 32 | 9 | 11.5 | 0.24 |
| 47 | 61 | -1 | 1 | 1 | 1 | -1 | 1 | 128 | 16 | 9 | 15.0 | 0.21 |
| 48 | 28 | 1 | 1 | 1 | 1 | -1 | 1 | 64 | 32 | 9 | 16.0 | 0.33 |
| 49 | 37 | -1 | -1 | -1 | -1 | 1 | 1 | 512 | 64 | 5 | 18.0 | 0.06 |
| 50 | 53 | 1 | -1 | -1 | -1 | 1 | 1 | 128 | 32 | 8 | 25.0 | 0.31 |
| 51 | 33 | -1 | 1 | -1 | -1 | 1 | 1 | 512 | 128 | 4 | 17.0 | 0.05 |
| 52 | 31 | 1 | 1 | -1 | -1 | 1 | 1 | 128 | 64 | 7 | 15.0 | 0.16 |
| 53 | 34 | -1 | -1 | 1 | -1 | 1 | 1 | 256 | 64 | 6 | 13.0 | 0.08 |
| 54 | 23 | 1 | -1 | 1 | -1 | 1 | 1 | 128 | 64 | 7 | 16.5 | 0.17 |
| 55 | 51 | -1 | 1 | 1 | -1 | 1 | 1 | 256 | 128 | 5 | 20.5 | 0.11 |
| 56 | 21 | 1 | 1 | 1 | -1 | 1 | 1 | 128 | 64 | 7 | 18.0 | 0.19 |
| 57 | 62 | -1 | -1 | -1 | 1 | 1 | 1 | 128 | 64 | 7 | 16.0 | 0.17 |
| 58 | 55 | 1 | -1 | -1 | 1 | 1 | 1 | 128 | 64 | 7 | 16.5 | 0.17 |
| 59 | 50 | -1 | 1 | -1 | 1 | 1 | 1 | 256 | 128 | 5 | 17.5 | 0.09 |
| 60 | 19 | 1 | 1 | -1 | 1 | 1 | 1 | 128 | 64 | 7 | 16.5 | 0.17 |
| 61 | 47 | -1 | -1 | 1 | 1 | 1 | 1 | 256 | 64 | 6 | 16.5 | 0.10 |
| 62 | 60 | 1 | -1 | 1 | 1 | 1 | 1 | 128 | 64 | 7 | 16.5 | 0.17 |
| 63 | 45 | -1 | 1 | 1 | 1 | 1 | 1 | 256 | 128 | 5 | 17.5 | 0.09 |
| 64 | 36 | 1 | 1 | 1 | 1 | 1 | 1 | 128 | 64 | 7 | 17.0 | 0.18 |
|  | | | |  |  |  |  |  |  |  |  |  |

* *C.a. Candida albicans; C.p. Candida parapsilosis*
